# Influence of d-Amino Acids in Beer on Formation of Uric Acid

**DOI:** 10.17113/ftb.57.03.19.6022

**Published:** 2019-09

**Authors:** Yuhe Kan, Zhikun Zhang, Kunhao Yang, Mengru Ti, Yongqi Ke, Li Wu, Jingkui Yang, Yujian He

**Affiliations:** 1 School of Chemical Sciences, University of Chinese Academy of Sciences, No.380 Huaibei Zhuang, Huairou District, 100049 Beijing, PR China; 2 State Key Laboratory of Natural and Biomimetic Drugs, School of Pharmaceutical Sciences, Peking University, 38 Xueyuan Road, Peking University, 100191 Beijing, PR China

**Keywords:** uric acid, beer, d-amino acid, hydroxyl radicals, DNA damage

## Abstract

Excessive intake of beer could increase serum uric acid levels, leading to high risk of gout, which was previously attributed to high purine content in beer. Recent reports that purine-rich vegetables and bean products do not cause higher uric acid levels do not support this theory. Why excessive intake of beer could increase a high risk of gout has been unclear. Other factors affecting the accumulation of uric acid in the blood have been explored. Beer contains relatively high levels of d-amino acids due to the racemization of l-amino acids induced by food processing. d-amino acid was catalyzed by d-amino acid oxidase to produce H_2_O_2_, which is further oxidized in the presence of Fe^2+^ to produce hydroxyl radicals, resulting in DNA damage and formation of a large amount of purine bases, which are oxidized to uric acid by a series of enzymes. Some food ingredients, such as vitamins and I^–^, prompt d-amino acids to form uric acid. d-amino acids in beer are one of the key factors responsible for the increase in uric acid levels. The biological response of d-amino acids could explain gout occurrence in beer drinkers.

## INTRODUCTION

As we know well, the high level of serum uric acid (H_2_U) *in vivo* causes hyperuricaemia (HUA), which is closely associated with the development and progression of gout, cardiovascular diseases, renal disease and tumour lysis syndrome (*1*–*3*). The higher serum H_2_U levels are also an independent indicator of these diseases (*4*). There is a great correlation between the H_2_U and daily diets (*5*, *6*). Recently, there have been reports that H_2_U levels are related to beer or beer and seafood intake (*7*, *8*), and it had been suggested that high level of purines in the beer can result in the increase in H_2_U in blood (*5*–*8*). However, it has been reported that purine-rich vegetables and bean products did not lead to higher H_2_U (*9*, *10*). Therefore, why beer drinkers get a higher risk of HUA should be explored.

Beer, as ‘liquid bread’, contains many amino acids in addition to the purines. Each amino acid has two isomers (l- and d-amino acid) (*11*). A relatively high d-amino acid (DAA) content in beer has been observed (*12*). Normally, l-amino acids (LAA) are the most crucial structural components in protein, and only rare DAAs are required for some normal biological functions (*e.g.* Ser) (*13*, *14*). The DAAs are maintained at relatively lower mass ratios (DAA/LAA<1.77%) in healthy biological tissues (*13*, *15*–*17*). Thus, DAAs in beer may be one of the factors that cause physiological dysfunction or some related diseases (such as HUA).

It has been reported that the DAA is catalyzed by d-amino acid oxidase (DAAO) to form NH_4_^+^, α-keto acid and H_2_O_2_ (*18*). The H_2_O_2_ further reacts with Fe^2+^ or Cu^+^ and is easily converted into highly reactive hydroxyl radical (˙OH) (*19*). Furthermore, vitamin B_2_ and I^–^ can improve the catalytic activity of DAAO during the hydrolysis of DAA. The reducing agent helps to reduce Fe^3+^ to Fe^2+^ or Cu^2+^ to Cu^+^, which is beneficial for the formation of active ˙OH. In fact, the concentrations of vitamins B_2_, C and E in beer are usually as high as 0.38, 30 and 3.0 mg/L, respectively (*20*, *21*), and seafood is usually rich in I^–^ (0.1 mmoL/kg), Fe^3+^ (364–1388 μg/g) and Cu^2+^ (17.1–143.9 μg/g) (*22*–*25*). This may provide all possible evidence for the more likely formation of ˙OH when consuming both beer and seafood. In addition, nucleic acids (DNA and RNA) are catalyzed to H_2_U through a series of enzymes, which, according to metabolic mechanisms, maintain the H_2_U in balance in blood under normal conditions. Yet, nucleic acids are damaged in the presence of ˙OH to form nucleotides and nucleosides, which are then converted to free purine bases that are necessary for H_2_U production (*26*). These data will provide a basis for links between DAA and H_2_U.

In view of previous arguments, abundance of purines was regarded as the most important factor in the formation of excess H_2_U. However, the possible linkage between the DAA and H_2_U has been ignored. In this report, the direct linkage between DAA in beer and H_2_U is based on molecular levels, and each metabolic process of DAA in beer was investigated during the conversion of DAA and H_2_U. A mechanism for the formation of free purines by nucleic acid damage caused by DAA is proposed, and the key role of DAA from beer in inducing the excessive H_2_U was found.

## MATERIALS AND METHODS

### Chemicals and material

All chemicals used are of analytical grade (>99.0%). l- and d-Ala were purchased from Tokyo Chemical Industry Co., Ltd. (Tokyo, Japan). l/d-isomers of Cys, Asn, Phe, Leu, His, Thr, Pro and Ser were from J&K Scientific Ltd. (Zhejiang, PR China). Sodium 3,5-dichloro-2-hydroxybenzenesulfonate (DHBS), adenosine deaminase (ADA, EC 3.5.4.4), phenyl isothiocyanate (PITC) and 4-aminoantipyine (4-AAP, 98%) were also from J&K Scientific Ltd. d-amino acid oxidase (DAAO, EC 1.4.3.3), purine nucleoside phosphorylase (PNPase, EC 2.4.2.1) and horseradish peroxidase (HRP, EC 1.11.1.7) were purchased from Sigma-Aldrich, Merck (St. Louis, MO, USA). Calf thymus DNA, riboflavin, xanthine oxidase (XOD, EC 1.2.3.2), vitamin C, vitamin E and catalase (CAT) were obtained from Solarbio Co., Ltd. (Beijing, PR China). Phosphate buffered saline (PBS) and Tris-acetate-EDTA (TAE) buffers were from Sangon Biotech Co., Ltd. (Shanghai, PR China). Methanol (chromatographic purity) was purchased from Siyou Co., Ltd. (Beijing, PR China). Agarose was obtained from Tiangen Co., Ltd. (Beijing, PR China). Triethylamine (TEA) was purchased from Shanghai Titan Co., Ltd. (Shanghai, PR China). Acetonitrile (ACN) was obtained from Sinopharm Chemical Reagent Co., Ltd. (Shanghai, PR China). Acetic acid was obtained from Beijing Chemical Works (Beijing, PR China). Yanjing beer (Yanjing Brewery Co., Ltd., Beijing, PR China), Tsingtao beer (Tsingtao Brewery Co., Ltd., Qingdao, PR China) and Harbin beer (Harbin Brewery Co., Ltd., Harbin, PR China) were purchased in a supermarket (Beijing, PR China). We confirm that experimental test was conducted within the warranty period. Ultrapure water was prepared using double distilled water (18 MΩ·cm).

### Assay of released H_2_O_2_ from the metabolism of DAA

The effect of different concentrations of l- and d-Ala on the formation of H_2_O_2_ catalyzed by DAAO was studied. The l- and d-Ala solutions at concentrations of 10, 1.0, 0.1, 0.01 and 0.001 mM were prepared with PBS buffer (pH=7.4, 0.01 M). Then, the chromogenic agent containing 228 U/L DAAO, 0.4 mM 4-AAP, 2.0 mM DHBS and 1500 U/L HRP was added into each of the above 3.0-mL solutions, and incubated at 37 °C for 30 min in digital constant temperature water bath pot (HH-4; Xinbao Instrument Factory, Guangzhou, PR China). DAA was oxidized by DAAO to generate H_2_O_2_, which was quantified by enzymatic coupling method and UV-Vis spectroscopy (*27*) using UV-2550 spectrophotometer (Shimadzu Corporation, Kyoto, Japan).

### Analysis of DNA damage by DAA

The effect of d-amino acid on the DNA was studied in PBS (0.01 M, pH=7.4). DNA damage was detected using DNA band diffusion assay on agarose gel (1%). The samples containing 0.1 mM Fe^2+^, 0.75 U/mL DAAO, 50 μg/mL DNA and different concentrations (0, 0.05, 0,1, 0,2, 0,4, 0.6 mM) of d-Ala were parallelly incubated at 37 °C for 30 min, and the degree of DNA damage was detected by agarose gel (1%) using electrophoresis system (JY600E universal electrophoresis apparatus; Beijing Junyi Electrophoresis Co., Ltd., Beijing, PR China). The reaction mixtures were terminated by the addition of 2 μL of loading buffer, and 10-μL aliquots were analysed by gel electrophoresis at 120 V for 1.5 h in the presence of a 1×TAE buffer using 1% agarose gel.

### Measurements of H_2_U formation induced by the presence of d-amino acid

The effect of DAA concentration on the formation of H_2_U was investigated. Samples containing DNA (50 μg/mL), Fe^2+^ (0.10 mM), different concentrations of d-Ala (0, 0.5, 1.0, 1.5, 2.0 and 2.5 mM) and DAAO with different activities (0. 0.05, 0.075, 0.10, 0.13, 0.13 U/mL) were prepared to simulate the formation of uric acid. The samples were incubated at 37 °C for 30 min, then CAT was added at 37 °C and kept at constant temperature for 30 min to remove H_2_O_2_ residues. Afterwards, the samples were heated at 70 °C for 15 min to inactivate CAT. To catalyze adenine and adenosine produced after DNA breaking into H_2_U and H_2_O_2_, PNPase (9.4 U/L), ADA (10.0 U/L) and XOD (5.0 U/L) were added and incubated at 37 °C for 30 min. All samples were incubated at 37 °C for 30 min in 2.7-mL reaction mixtures containing 1500 U/L HRP, 0.4 mM 4-AAP and 2.0 mM DHBS. The absorbance of H_2_U was indirectly detected at 512 nm by UV-Vis spectroscopy (UV-2550; Shimadzu Corporation).

### The relationship between DAA in beer and the formation of H_2_U

Beer samples of 50 μL were dried in vacuum drying oven (DZF-6050; Beijing Land and Technology Co., Ltd., Beijing, PR China). The obtained dry powders were mixed with 0.75 U/mL DAAO and dissolved in 100 μL water, and then incubated at 37 °C for 30 min, followed by drying in vacuum drying oven. The d- and l-amino acids were determined based on the method of Cohen *et al*. (*28*). The derivatized amino acids were determined by HPLC (LC-20AT; Shimadzu Corporation), equipped with Eclipse XDB-C18 column (250 mm×9.4 mm i.d., particle size 5 μM). The gradient elution was performed at 0−10 min mobile phase A 90% (0.14 mol/L CH_3_COONa, 0.5 mol/L TEA, pH=6.40±0.05), 10−11 min mobile phase B 12.5% (100% ACN, pH=7.5, and ultrapure water, 60:40 by volume) and 11–30 min mobile phase A 90%. Injection volume was 5 μL, the column was maintained at 25 °C, detection wavelength was 254 nm. The flow rate of the mobile phase was 0.8 mL/min, the control samples were prepared by dissolving dry powders in 100 μL water without DAAO. The total DAA content in beer samples was calculated by comparing the peak areas of standard amino acid and DAA in the beer at same retention time. Each test was done in triplicate and the content of DAA in the beer was expressed as mean value±standard deviation.

The samples of 0.50 and 1.0 mL of Yanjing beer were pipetted into individual tubes and dried in vacuum drying oven (DZF-6050; Beijing Land and Technology Co., Ltd.) at 45 °C to remove the ethanol. The dry powers were dissolved in 1.0 mL water, and then the PNPase, ADA and XOD were added to the two samples to remove the adenine and adenosine, thereby eliminating their interference. The interferences were detected by enzymatic coupling method and UV-Vis (*27*) spectroscopy (UV-2550; Shimadzu Corporation). CAT was added to the above-mentioned samples at 37 °C and kept at constant temperature for 30 min to remove H_2_O_2_, and then the obtained solution was maintained at 70 °C for 15 min to inactivate CAT, followed by drying again at 45 °C. Control samples containing 50 μg/mL DNA without adenine and adenosine and test group containing 0.75 U/mL DAAO, 0.10 mM Fe^2+^ and 50 μg/mL DNA were prepared. These samples were incubated at 37 °C for 30 min, and then CAT was added at 37 °C and kept at constant temperature for 30 min to remove H_2_O_2_ residues. Afterwards, the samples were heated at 70 °C for 15 min to inactivate CAT, and then PNPase, ADA and XOD with final activities of 9.4, 10.0 and 5.0 U/L respectively were added and the samples were incubated for 30 min at 37 °C. All samples were kept at 37 °C for 30 min in 2.7-mL reaction mixtures containing 1500 U/L HRP, 0.4 mM 4-AAP and 2.0 mM DHBS. The absorbance was read at 512 nm using UV-Vis spectrophotometer (UV-2550; Shimadzu Corporation).

### The assay of the role of vitamins and iodide ions on the formation of uric acid

The effect of vitamin B_2_ on the activity of DAAO was studied. The d-Ala (final concentration 2.8 mM) and DAAO (final concentration 0.1 U/mL) were mixed with different concentrations (0, 0.50, 0.75, 1.0 mg/L) of vitamin B_2_ in 3.0 mL of PBS buffer. The samples were incubated at 37 °C for 1 h, and then the absorbance of the pyruvic acid was measured at 260 nm by UV-Vis spectrophotometer (UV-2550; Shimadzu Corporation) to evaluate the effect of vitamin B_2_ on the activity of DAAO.

The effects of vitamins C and E on the formation of H_2_U were studied. The samples containing 0.1 mM Fe^3+^, 0.75 mg/L vitamin E or vitamin C, 50 μg/mL DNA, 0.1 mM DAA and 0.75 U/mL DAAO were incubated at 37 °C for 40 min, followed by gel electrophoresis (JY600E universal electrophoresis apparatus; Beijing Junyi Electrophoresis Co., Ltd.) at 120 V for 1.5 h on 1% agarose gel.

The effects of I^–^ and Fe^3+^ on the formation of H_2_U were estimated by the determination of ˙OH. The highly reactive ˙OH reacts with Phe and produces *o*-, *m*-, and *p*-Tyr (*29*, *30*). The mixture (pH=7.4) containing 10 mM Phe, 0.1 mM Fe^3+^ (Fe^3+^–EDTA complex), 0.1 mM DAA and 0.75 U/mL DAAO was incubated for 40 min at 37 °C. The concentration of ˙OH in the mixture was determined by HPLC (LC-20AT; Shimadzu Corporation, with C18 column, 9.4 mm×250 mm), using isocratic elution consisting of 250 mM KH_2_PO_4_/H_3_PO_4_ (pH=3.01) with 5% methanol (by volume) at the flow rate of 0.8 mL/min. The absorbance of the samples was evaluated at 274 nm by UV- -2550 spectrophotometer (Shimadzu Corporation).

DNA samples (50 μg/mL) were treated with I^–^ (0.10 mM), Fe^3+^–EDTA (0.10 mM), d-Ala (0, 20, 40, 60, 80, 100 mM) and DAAO (pH=7.4, 0.75 U/mL). The degree of DNA damage was determined using DNA band diffusion assay on agarose gel (1%).

The effect of the racemization of LAA on the formation of H_2_U was studied. As we know, the seafood contains abundance of metal ions (*e.g.* Fe^3+^ and Cu^2+^) (*11*). LAA can be racemized by heating or adding metal ions (*17*). The sample containing 0.03 mmol/L l-Ala, Cu^2+^ and water to give a total reaction volume of 1 mL (pH=7.0) was prepared and incubated at 100 °C for 3 h. The Cu^2+^ to l-Ala ratios were 0, 1:8, 1:4 and 1:2. The racemization of l-Ala were detected by circular dichroism spectrophotometer (Jasco-815; Jasco Corporation, Tokyo, Japan) in the 190–240 nm wavelength region at room temperature.

## RESULTS AND DISCUSSION

### The effect of DAA in aqueous solution on the formation of uric acid

**Fig. 1:**
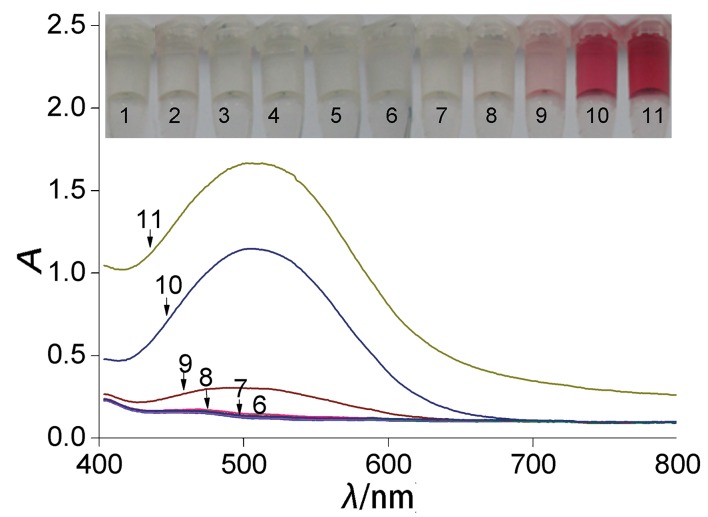
Chromogenic reaction of different concentrations of l/d-amino acids and absorbance in the visible spectrum of the reaction mixtures of l/d-Ala oxidized by d-amino acid oxidase. Samples 1-6: *c*(l-Ala)=0, 0.001, 0.01, 0.1, 1.0 and 10 mM, respectively, samples 7-11: *c*(d-Ala)=0.001, 0.01, 0.1, 1.0 and 10 mM, respectively

As previously reported, H_2_O_2_ was one of metabolites of d-isomeric amino acids in the presence of d-amino acid oxidase (DAAO) due to stereospecificity of the deamination of amino acids (*18*). DAA was oxidized and deaminated to produce H_2_O_2_, which reacted with 4-AAP and DHBS to produce red quinone imine dye in the presence of HRP. The H_2_O_2_, as a product of DAA degradation, was first measured by enzymatic coupling method and UV-Vis spectroscopy. As shown in Fig. 1, samples 1–6 with different concentrations of l-Ala were colourless, which indicated that l-Ala was not catalyzed to H_2_O_2_ by DAAO. In contrast, samples 7–11 with different concentrations of d-Ala were striking red, and the colour became darker with the increase of the concentrations of d-Ala. These results confirm that H_2_O_2_ is produced by DAAO from DAA rather than LAA, and that it can be detected by enzymatic coupling in combination with UV-Vis spectroscopy, which has high sensitivity and specificity (0.1 mM of DAA). Our results draw the same conclusions as our previous research (*27*), where colour of reaction sample was significantly darker with concentrations of DAA ranging from 0.1 to 10 mM.

It has long been known that the reaction involving H_2_O_2_ with redox-active iron or copper (*i.e.* Fenton reaction) produces ˙OH, leading to DNA damage (*19*, *31*). To further assess the effects of H_2_O_2_ derived from DAA on the production of ˙OH and DNA damage, we performed the DNA damage studies with d-Ala, DAAO and Fe^2+^. With the increase of the concentration of d-Ala the band became more diffuse at constant concentrations of DAAO and Fe^2+^ (Fig. S1), indicating that DAA can act as an effective disruptor for DNA in the presence of DAAO and Fe^2+^. Other research on DNA damage with salmon sperm DNA and pBluescript K^+^ plasmid had proven that the presence of hydroxyl radicals leads to DNA breaks (*19*).

Our final attention was focused on the formation of H_2_U from DAA, although we confirmed that H_2_O_2_ was produced from DAA in the presence of DAAO, followed by the damage of DNA in the presence of redox-active iron, caused by the production of ˙OH. A research reported that the produced hydrogen peroxide was able to quench the quantum dot (QD) fluorescence, which was proportional to uric acid concentration (*32*). Thus, the H_2_U could be determined by detection of H_2_O_2_ using chromogenic agent, because the new products of H_2_O_2_ indirectly show the product of H_2_U in the process of converting nucleic acids into uric acid when the H_2_O_2_ formed in the first step is removed by CAT.

**Fig. 2:**
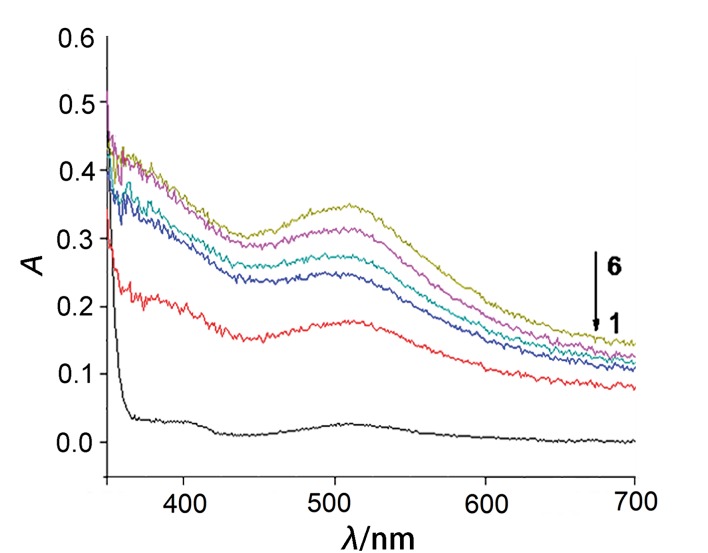
Influence of d-amino acid concentrations on uric acid formation. Spectra 1-6: *c*(d-Ala)=0, 0.5, 1.0, 1.5, 2.0 and 2.5 mM

The H_2_U concentration increased twice when the concentration of d-Ala was in the range from 0.5 to 2.5 mM (Fig. 2), which suggested that DAA can produce ˙OH in the presence of DAAO and Fe^2+^ and damage DNA, leading to excessive formation of H_2_U. The results indicate that during oxidation of DAA purine and pyrimidine bases are formed, and purine bases can be oxidized by xanthine oxidase to H_2_U.

### The effect of DAA in beer on the formation of uric acid

To investigate the important role of DAA in beer in inducing H_2_U, DAA content in the solution of beer samples (Yanjing, Tsingtao and Harbin) was determined by HPLC and compared with standard amino acids (Cys 0.75, Ser 1.00, Asn 0.54, Phe 1.00, His 0.65, Thr 1.80, Ala 1.89, Pro 0.90 and Arg 0.80 mM). PITC reacted with amino acids to form stable phenylthiocarbamyl derivatives (*28*), which can be effectively used to analyze and quantify amino acids in beer. As shown in Fig. S2, eight types of amino acids in beer were observed at different retention times. The total contents of amino acids in beer and the remaining LAAs after catalysis of all DAAs by DAAO were determined by HPLC. The content of DAAs in beer was detected according to the difference of HPLC peak areas between the samples treated with DAAO and control ones (without treatment) and calculated by comparing this difference with the amino acid standard. The concentrations of DAA in Yanjing, Tsingtao and Harbin beer were (0.80±0.04), (0.34±0.02) and (0.29±0.01) mM, respectively (data not shown).

The DAA in beer may be produced from microbial metabolites or by racemization of free and protein-bound LAA during food processing (*12*). We used circular dichroism spectrophotometer to detect active chiral molecules, such as LAA/DAA. The effect of temperature and metal ions on the racemization of l-Ala was studied based on the simulation of beer processing. During cooking or heating, Cu^2+^ can catalyze the racemization of l-Ala at pH=7 and 100 °C. Racemization of l-Ala increased with the increase of the l-Ala to Cu^2+^ ratio, which proves that the rate of racemization depends on the Cu^2+^ concentration and it could be as high as 40.1% when the molar ratio of l-Ala to Cu^2+^ was 2:1 (Fig. S3). Erbe and Brückner (*33*) detected d-Pro in matured wine vinegar by chiral gas chromatography, which proved that relatively high amounts of d-Pro were mainly attributed to the Maillard reaction. Therefore, the presence of DAA in beer could be attributed to Maillard reaction caused by heating during beer production.

Based on our studies on DAA in aqueous solution, we know that DAA could be used as a potent DNA-damaging agent in the presence of DAAO and Fe^2+^, which induced the formation of H_2_U. To prove that DAA can induce DNA damage, we excluded the possible factors including ethanol, adenine and adenosine (*5*–*8*). Adenine and adenosine in the beer solution were effectively removed using ADA, PNPase and XOD, and the ethanol was also removed using vacuum drying at 45 °C. Detection of H_2_O_2_ indirectly proved the production of H_2_U, because H_2_O_2_ and H_2_U were formed together during metabolic degradation of adenine and adenosine.

**Fig. 3:**
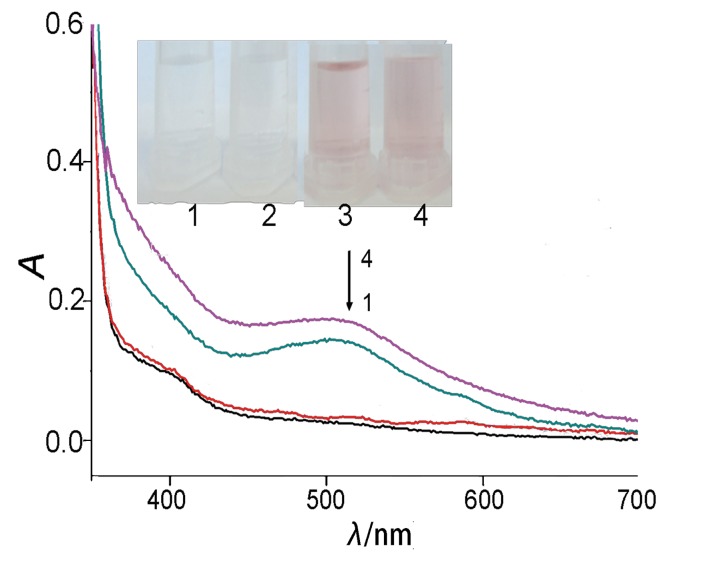
Chromogenic reaction and the absorbance in the visible spectrum of the samples treated with ADA and PNPase. Test tubes and spectra: 1 and 2: *V*(Yanjing beer)=0.5 and 1.0 mL with the addition of d-amino acid oxidase, 3 and 4: *V*(Yanjing beer)=0.5 and 1.0 mL with the addition of Fe^2+^ and d-amino acid oxidase. ADA=10.0 U/L, PNPase=9.4 U/L

Fig. 3 shows that there was no colour change of samples 1 and 2 with different volumes of beer when they were treated with ADA and PNPase, indicating that adenine and adenosine could be completely removed from the beer. Adenine and adenosine in samples 3 and 4 were removed by the same method, and incubated in the presence of Fe^2+^ and DAAO. Values of *A*_512 nm_ of samples 3 and 4 increased with the increase of the volumes of the beer, which showed that the concentration of H_2_U increased by 27% when the volumes of beer increased twice (Fig. 3). This confirmed that the mixture of beer with DNA, Fe^2+^ and DAAO could cause DNA damage and form H_2_U. Blood accounts for about 8% (by mass) of the human body (*34*). The average body mass of the adult is 75 kg, and the average blood volume is 60 dL. Normal levels of H_2_U in blood are 2.18–7.0 mg/dL (*30*), and if increased after the consumption of large volumes of beer, it could easily result in gout.

### The effect of vitamins and iodide ions on the formation of uric acid induced by DAA

**Fig. 4:**
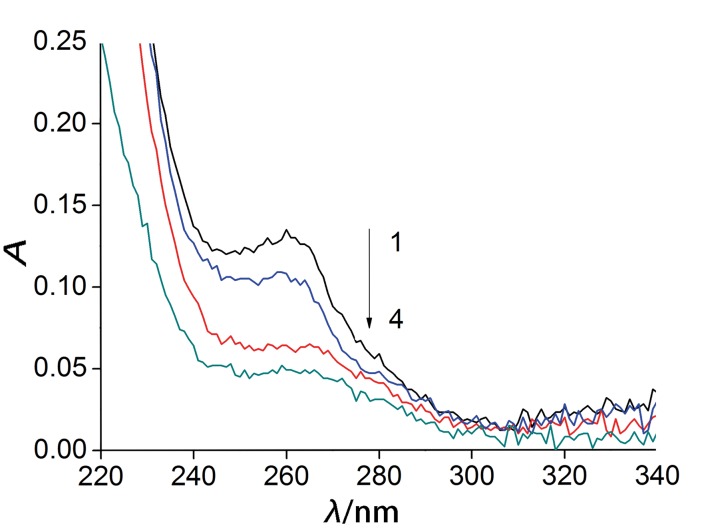
The effect of riboflavin (vitamin B2) on d-amino acid oxidase activity. Spectra 1-4: *c*(riboflavin)=1.00, 0.75, 0.50 and 0 mM

A previous study reported that vitamin B_2_ content in beer was in the range of 0.5–1.0 μM, and the active form of vitamin B_2_ is a coenzyme of DAAO (*21*). In the presence of DAAO, d-Ala was oxidized to pyruvic acid, which has strong UV absorption. We thus evaluated the effect of riboflavin on DAAO by quantification of pyruvic acid. Fig. 4 shows that the concentration of pyruvic acid increased with the increase of riboflavin concentration from 0 to 1.0 mM, which indicated that DAAO activity increased up to 1.7 times due to the presence of riboflavin.

**Fig. 5:**
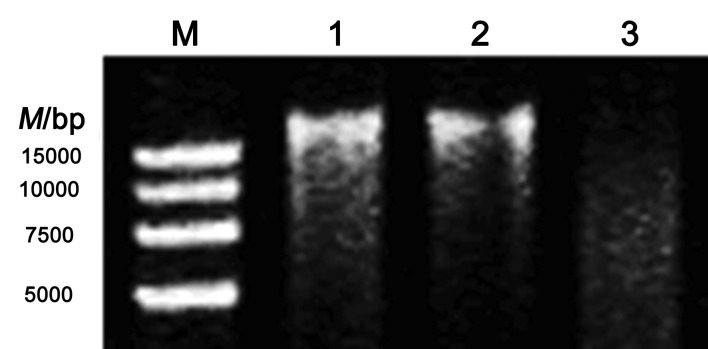
DNA damage induced by 0.8 mM d-Ala with 0.2 mM Fe^3+^ and 0.8 U/mL d-amino acid oxidase with or without vitamin E using 1% agarose gel. M=molecular marker, lane 1=DNA sample, lane 2=DNA damage without vitamin E, lane 3=DNA damage in the presence of 0.2 mM vitamin E

Vitamin E, as an ingredient of beer, can promote DNA damage induced by H_2_O_2_ and Fe^3+^ or Cu^2+^ ions *via* Fenton reaction (*35*). H_2_O_2_ produced by DAA and DAAO could more easily react with Fe^2+^ than with Cu^+^ to form ˙OH, and thus we studied the reduction of Fe^3+^ to Fe^2+^ by vitamin E, which caused the DNA damage in the presence of DAA and DAAO. Fig. 5 shows the DNA damage in the presence or absence of vitamin E. DNA band was slightly dispersed in the presence of 0.2 mM vitamin E in lane 3 and it still existed in the absence of vitamin E in lane 2, suggesting that vitamin E accelerated DNA damage with DAA and DAAO. Also, vitamin C could directly react with H_2_O_2_ to generate ˙OH and damage DNA even without the assistance of metal ions, since the reduction ability of vitamin C was stronger than of vitamin E (*36*). Thus, vitamin C in beer can promote formation of H_2_U.

**Fig. 6:**
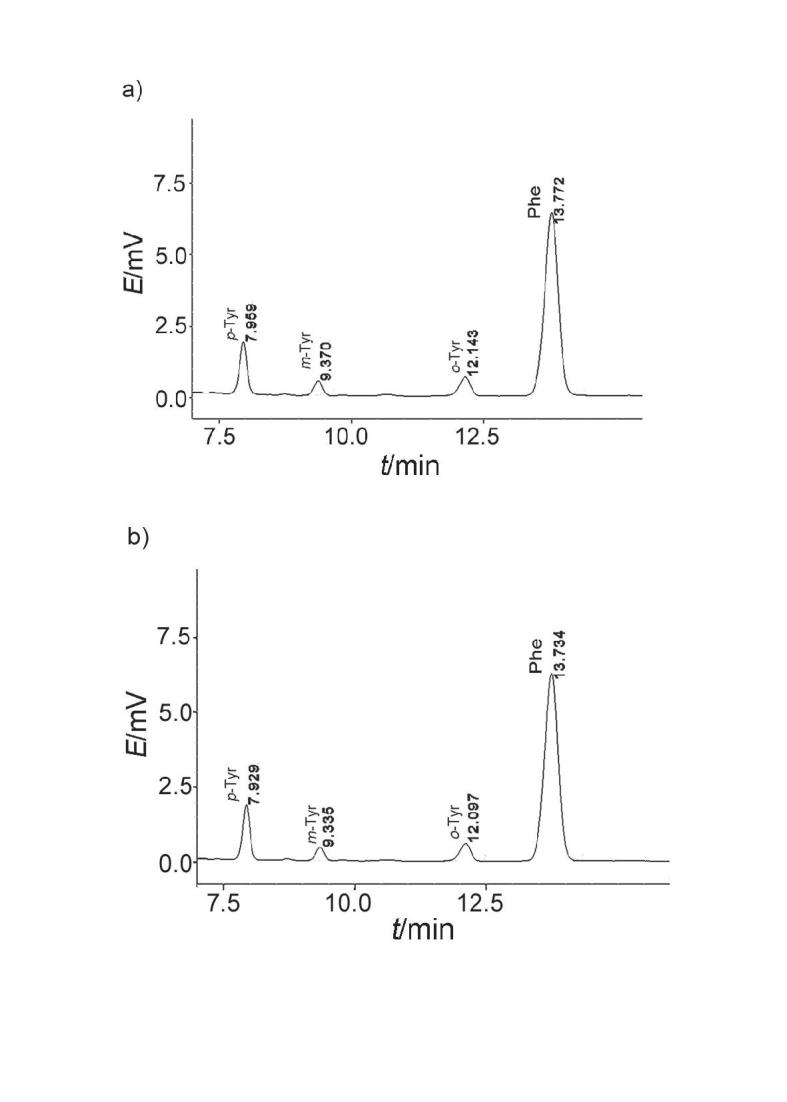
Consumption of ˙OH by phenylalanine (10 mM) in the presence of 0.1 mM I^–^, 0.75 U/mL d-amino acid oxidase, 0.1 mM d-Ala and a) 0.1 mM Fe^3+^ or b) 0.1 mM Fe^3+^–EDTA

Just as importantly, I^–^ exists in daily diet together with vitamins, and its impact on DNA damage induced by DAA should not be neglected because I^–^ has strong ability to reduce Fe^3+^ and Cu^2+^. Several isomeric tyrosines can be produced in the reaction of phenylalanine with ˙OH and total amount of products was proportional to the amount of ˙OH in the system (*29*, *30*). Fig. 6 shows that three peak areas of the products (*p*-, *m*- and *o*-Tyr) in the presence of Fe^3+^ were 18054, 5854 and 9944, respectively (Fig. 6a) and the corresponding peak areas for the products in the presence of EDTA were 17881, 5032 and 8719, respectively (Fig. 6b). The retention times of 7.95, 9.37 and 12.14 min corresponding to *p*-, *m*- and *o*-tyrosine were generated from phenylalanine and ˙OH. The results indirectly show that the formation of ˙OH from 0.1 mM d-Ala in PBS containing 0.1 mM I^–^ and 0.75 U/mL DAAO was better in the presence of Fe^3+^ than of Fe^3+^-EDTA.

Actually, chelation by Fe^3+^–EDTA decreases the formation of ˙OH, while the production of reduction product (Fe^2+^) by I^–^ promotes the formation of ˙OH. In addition, DNA damage induced by 0.1 mM d-Ala in PBS containing different concentrations of I^–^ and 0.75 U/mL DAAO in the presence of Fe^3+^ (lanes 1–5) or Fe^3+^–EDTA (lanes 6–10) was further investigated, as shown in Fig. S4. Lanes 1–5 show DNA damage levels of 30.0, 47.0, 50.6, 51.1 and 51.1%, respectively. However, when Fe^3+^ was chelated by EDTA, lanes 6–10 showed the DNA damage levels of 33.3, 33.4, 33.4, 33.5 and 33.6%, respectively. This confirms that the complex of Fe^3+^–EDTA can prevent production of reduction product (Fe^2+^) by I^–^, thereby inhibiting the conversion of H_2_O_2_ into ˙OH and reducing DNA damage.

Gout occurs as a result of H_2_U accumulation in the blood, caused by purine metabolism disorder. Exogenous (about 20%) and endogenous (about 80%) purines are present in the body. The former originate from daily diet, and the latter are formed by the oxidation of nucleic acids, which mainly lead to the formation of purine. Previous surveys have shown that excessive intake of beer can increase the risk of gout, which could be attributed to high level of purines in beer (*5*–*8*). However, recent reports have shown that the content of H_2_U in the serum remains the same or even decreases when purine-rich beans and vegetables are being consumed (*9*, *10*). Factors other than purine-rich diet can also promote the accumulation of uric acid in the blood. In the metabolic pathway of purine, the oxidization of nucleic acid leads primarily to the production of purines, but further oxidation gives a balanced amount of H_2_U as a final product. Once hydrogen peroxide and free radicals are produced, the nucleic acids are oxidized faster and produce more purines, which leads to the increase of H_2_U concentration. The DAA in food is absorbed in liver, kidney, or through the stomach and intestine, and it can be oxidized and deaminated to NH_4_^+^, α-keto acid and H_2_O_2_, which can react with Fe^2+^ or Cu^+^ to generate highly reactive ˙OH (*18*). Therefore, we propose that there is a direct linkage between DAA and H_2_U concentrations.

In aqueous solution, DAA produces H_2_O_2_ in the presence of DAAO, which could be monitored by the chromogenic reaction. Assay of DNA damage shows that oxidation of nucleic acids is related to DAA content, which proves that relatively high content of DAA increases the degree of DNA damage. Beer, as a fermented drink, has a relatively high content of DAA. Next, we have investigated the influence of DAA presence in the beer on the formation of H_2_U, a key factor that induces gout. ADA and PNPase removed purines in order to eliminate their interference, while H_2_U was product of the reaction of beer powder with DAAO and Fe^2+^.

In addition, our experiments show that vitamins and I^–^ can promote the formation of H_2_U by DAA, which confirms the key role of DAA in the formation of H_2_U in beer. Therefore, the molecular mechanism by which DAA forms H_2_U is summarized. The DAA catalyzed by DAAO is oxidized to H_2_O_2_, which further reacts with Fe^2+^ or Cu^+^ to produce highly reactive ˙OH. The nucleic acid is damaged by ˙OH and produces a large amount of hydrazine, which is oxidized to H_2_U by a series of enzymes. The study explains why excessive intake of beer can cause gout, and purine-rich foods are one of the most important factors leading to gout.

## CONCLUSIONS

Here we discussed the effect of d-amino acid (DAA) in beer on the formation of serum uric acid (H_2_U) and found that DAA could be oxidized to H_2_O_2_ by d-amino acid oxidase (DAAO). Further oxidization gave highly active hydroxyl radicals, which would accelerate the nucleic acid metabolism to purines and elevate H_2_U level, resulting in gout. We conclude that DAAs in beer have a key role in inducing the increase of H_2_U, and some food ingredients, such as vitamins and iodide ions, intensify the effect.
